# Comparative evaluation of set-level techniques in predictive classification of gene expression samples

**DOI:** 10.1186/1471-2105-13-S10-S15

**Published:** 2012-06-25

**Authors:** Matěj Holec, Jiří Kléma, Filip Železný, Jakub Tolar

**Affiliations:** 1 Faculty of Electrical Engineering, Czech Technical University in Prague, Prague, 166 27, Czech Republic; 2 Department of Pediatrics, University of Minnesota, Minneapolis, 55454, USA

## Abstract

**Background:**

Analysis of gene expression data in terms of a priori-defined gene sets has recently received significant attention as this approach typically yields more compact and interpretable results than those produced by traditional methods that rely on individual genes. The set-level strategy can also be adopted with similar benefits in predictive classification tasks accomplished with machine learning algorithms. Initial studies into the predictive performance of set-level classifiers have yielded rather controversial results. The goal of this study is to provide a more conclusive evaluation by testing various components of the set-level framework within a large collection of machine learning experiments.

**Results:**

Genuine curated gene sets constitute better features for classification than sets assembled without biological relevance. For identifying the best gene sets for classification, the Global test outperforms the gene-set methods GSEA and SAM-GS as well as two generic feature selection methods. To aggregate expressions of genes into a feature value, the singular value decomposition (SVD) method as well as the SetSig technique improve on simple arithmetic averaging. Set-level classifiers learned with 10 features constituted by the Global test slightly outperform baseline gene-level classifiers learned with all original data features although they are slightly less accurate than gene-level classifiers learned with a prior feature-selection step.

**Conclusion:**

Set-level classifiers do not boost predictive accuracy, however, they do achieve competitive accuracy if learned with the right combination of ingredients.

**Availability:**

Open-source, publicly available software was used for classifier learning and testing. The gene expression datasets and the gene set database used are also publicly available. The full tabulation of experimental results is available at http://ida.felk.cvut.cz/CESLT.

## Background

*Set-level *techniques have recently attracted significant attention in the area of gene expression data analysis [1-7]. Whereas in traditional analysis approaches one typically seeks individual genes differentially expressed across sample classes (e.g. cancerous vs. control), in the set-level approach one aims to identify entire sets of genes that are significant, e.g. in the sense that they contain an unexpectedly large number of differentially expressed genes. The gene sets considered for significance testing are defined prior to analysis, using appropriate biological background knowledge. For example, a defined gene set may contain genes acting in a given cellular pathway or annotated by a specific term of the gene ontology. The main advantage brought by set-level analysis is the compactness and improved interpretability of analysis results due to the smaller number of the set-level units compared to the number of genes, and more background knowledge available to such units. Indeed, the long lists of differentially expressed genes characteristic of traditional expression analysis are replaced by shorter lists of more informative units corresponding to actual biological processes.

*Predictive classification *[8] is a form of data analysis going beyond the mere identification of differentially expressed units. Here, units deemed significant for the discrimination between sample classes are assembled into formal models prescribing how to classify new samples that contain yet unknown class labels. Predictive classification techniques are thus especially relevant to diagnostic tasks and as such have been explored since very early studies on microarray data analysis [9]. Predictive models are usually constructed by supervised machine learning algorithms [8,10] that automatically discover patterns among samples with already available labels (so-called *training samples*). Learned classifiers may take diverse forms ranging from geometrically conceived models such as *Support Vector Machines *[11], which have been especially popular in the gene expression domain, to symbolic models such as logical rules or decision trees that have also been applied in this area [12-14].

The combination of set-level techniques with predictive classification has been suggested [7,15,16] or applied in specific ways [4,17-20] in previous studies, however, a focused exploration of the strategy has commenced only recently [21,22].

The set-level framework is adopted in predictive classification as follows. Sample features originally bearing the (normalized) expressions of individual genes are replaced by features corresponding to gene sets. Each such feature aggregates the expressions of the genes contained in the corresponding set into a single real value; in the simplest case, it may be the average expression of the contained genes. The expression samples are then presented to the learning algorithm in terms of these derived, set-level features. The main motivation for extending the set-level framework to the machine learning setting is again the interpretability of results. Informally, classifiers learned using set-level features acquire forms such as "predict cancer if pathway P1 is active and pathway P2 is not" (where *activity *refers to aggregated expressions of the member genes). In contrast, classifiers learned in the standard setting derive predictions from expressions of individual genes; it is usually difficult to find relationships among the genes involved in such a classifier and to interpret the latter in terms of biological processes.

Lifting features to the set level incurs a significant compression of the training data since the number of considered gene sets is typically much smaller than the number of interrogated genes. This compression raises the natural question whether relevant information is lost in the transformation, and whether the augmented interpretability will be outweighed by compromised predictive accuracy. On the other hand, reducing the number of sample features may mitigate the risk of overfitting and thus, conversely, contribute to higher accuracy. In machine learning terms, reformulation of data samples through set-level features increases the *bias *and decreases the *variance *of the learning process [8]. An objective of this study is to assess experimentally the combined effect of the two antagonistic factors on the resulting predictive accuracy.

Another aspect of transforming features to the set level is that biological background knowledge is channeled into learning through the prior definitions of biologically plausible gene sets. Among the goals of this study is to assess how significantly such background knowledge contributes to the performance of learned classifiers. We do this assessment by comparing classification accuracy achieved with genuine curated gene sets against that obtained with gene sets identical to the latter in number and sizes, yet lacking any biological relevance. We also investigate patterns distinguishing genuine gene sets particularly useful for classification from those less useful.

A further objective is to evaluate--from the machine learning perspective--statistical techniques proposed recently in the research on set-level gene expression analysis. These are the Gene Set Enrichment Analysis (GSEA) method [1], the SAM-GS algorithm [3] and a technique known as the Global test [2]. Informally, they rank a given collection of gene sets according to their correlation with phenotype classes. The methods naturally translate into the machine learning context in that they facilitate feature selection [23], i.e. they are used to determine which gene sets should be provided as sample features to the learning algorithm. We experimentally verify whether these methods work reasonably in the classification setting, i.e. whether learning algorithms produce better classifiers from gene sets ranked high by the mentioned methods than from those ranking lower. We investigate classification conducted with a single selected gene set as well as with a batch of high ranking sets. Furthermore, we test how the three gene-set-specific methods compare to some generic feature selection heuristics (information gain and support vector machine with recursive feature extraction) known from machine learning.

To use a machine learning algorithm, a unique value for each feature of each training sample must be established. Set-level features correspond to multiple expressions and these must therefore be aggregated. We comparatively evaluate three aggregation options. The first (AVG) simply averages the expressions of the involved genes. The value assigned to a sample and a gene set is independent of other samples and classes. The other two, more sophisticated, methods (SVD, SetSig) rely respectively on the singular value decomposition principle [7] and the so-called gene set signatures [22]. In the latter two approaches, the value assigned to a given sample and a gene set depends also on expressions measured in other samples. Let us return to the initial experimental question concerned with how the final predictive accuracy is influenced by the training data compression incurred by reformulating features to the set level. As follows from the above, two factors contribute to this compression: selection (not every gene from the original sample representation is a member of a gene set used in the set-level representation, i.e. some interrogated genes become ignored) and aggregation (for every gene set in the set-level representation, expressions of all its members are aggregated into a single value). We quantify the effects of these factors on predictive accuracy. Regarding selection, we experiment with set-level representations based on 10 best gene sets and 1 best gene set, respectively, with both numbers chosen ad-hoc. The two options are applied with all three selection methods (GSEA, SAM-GS, Global). We compare the obtained accuracy to the baseline case where all individual genes are provided as features to the learning algorithm, and to an augmented baseline case where a prior feature-selection step is taken using the information gain heuristic. For each of the selection cases, we further evaluate the contribution of the aggregation factor. This evaluation is done by comparing all the three aggregation mechanisms (AVG, SVD, SetSig) to the control case where no aggregation is performed at all; in this case, individual genes combined from the selected gene groups act as features.

The key contribution of the present study is thus a thorough experimental evaluation of a number of aspects and methods of the set-level strategy employed in the machine learning context, entailing the reformulation of various, independently published relevant techniques into a unified framework. Such a contribution is important both due to the current state of the art in microarray data analysis, wherein according to the review [24], *the need for thoroughly evaluating existing techniques currently seems to outweigh the need to develop new techniques*, and specifically due to the inconclusive results of previous, less extensive studies indicating both superiority (e.g. [20]) and inferiority (Section 4 in [22]) of the set-level approach to classificatory machine learning, with respect to the accuracy achievable by the baseline gene-level approach.

Our contributions are, however, also significant beyond the machine learning scope. In the general area of set-level expression analysis, it is undoubtedly important to establish a performance ranking of the various statistical techniques for the identification of significant gene sets in class-labeled expression data. This is made difficult by the lack of an unquestionable ranking criterion--there is in general no ground truth stipulating which gene sets should indeed be identified by the tested algorithms. The typical approach embraced by comparative studies such as [3] is thus to appeal to intuition (e.g. *the p53 pathway should be identified in p53-gene mutation data*). However legitimate such arguments are, evaluations based on them are obviously limited in generality and objectivity. We propose that the predictive classification setting supported by the cross-validation procedure for unbiased accuracy estimation, as adopted in this paper, represents exactly such a needed framework enabling objective comparative assessment of gene set selection techniques. In this framework, results of gene set selection are deemed good if the selected gene sets allow accurate classification of new samples. Through cross-validation, the accuracy can be estimated in an unbiased manner.

## Main results

**Figure 1:**
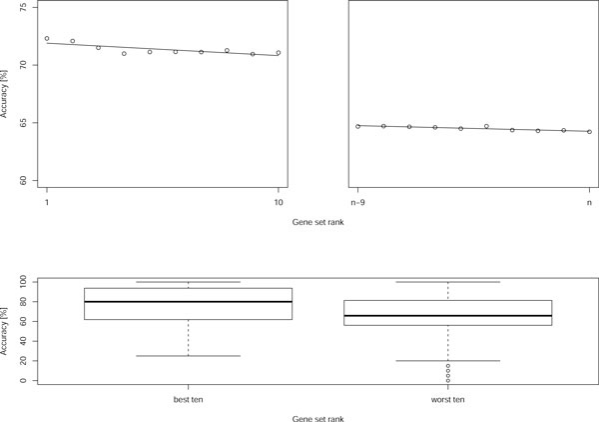
**Accuracy decay**. The top panels show the plots for the average accuracy of Factor 3 alternatives ranging 1 to 10, and n−9 to n. Average predictive accuracy tends to fall as lower-ranking gene sets are used to constitute features (see text for details). The trend lines shown in the top panels are the ones minimizing the residual least squares. The bottom panel gives the accuracy boxplot for the batch experiments. 10 highest-ranking and the 10 lowest-ranking (respectively) gene sets are combined to produce a feature set for learning. Again, the dominance of the former in terms of accuracy is obvious. Each point in the top panels and each box plot in the bottom panel follows from 16,000 learning experiments.

We first verified whether gene sets ranked high by the established set-level analysis methods (GSEA, SAM-GS, Global) indeed lead to construction of better classifiers by machine learning algorithms, i.e. we investigated how classification accuracy depends on Factor 3 in Table 1. In the top panel of Figure 1, we plot the average accuracy for Factor 3 alternatives ranging 1 to 10 (top 10 gene sets), and *n *− 9 to *n *(bottom 10). The trend line fitted by the least squares method shows a clear decay of accuracy as lower-ranking sets are used for learning. The bottom panel corresponds to Factor 3 values 1:10 (left) and *n *− 9 : *n *(right) corresponding to the situations where the 10 top-ranking and the 10 bottom-ranking (respectively) gene sets are combined to produce a feature set for learning. Again, the dominance of the former in terms of accuracy is obvious.

**Table 1:** Factors

Analyzed factors	*Alternatives*	*#Alts*
*1. Gene sets (Sec.)*	Genuine, Random	2
*2. Ranking algo (Sec.)*	GSEA, SAM-GS, Global	3
3. Set(s) forming features*	1, 2, ... 10,*n *- 9, *n *- 8,...*n*,1:10, *n *- 9 : *n*	22
*4. Aggregation (Sec.)*	SVD, AVG, SetSig, None	4

*Product*		528

**Auxiliary factors**	***Alternatives***	**#*Alts***

*5. Learning algo (Sec.)*	svm, 1-nn, 3-nn, nb, dt	5
*6. Dataset (Sec.)*	*d*_1 _... *d*_30_	30
*7. Testing Fold*	*f*_1 _... *f*_10_	10

*Product*		1500

**Figure 2:**
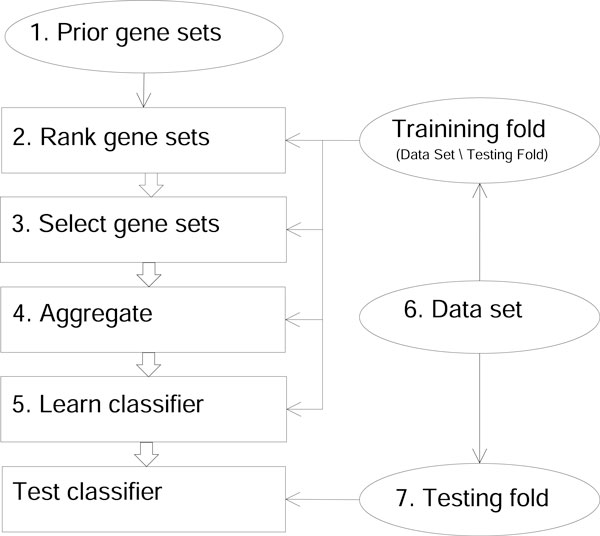
**Workflow**. The workflow of a set-level learning experiment conducted multiple times with varying alternatives in the numbered steps. For compatibility with the learned classifier, testing fold samples are also reformulated to the set level. The reformulation is done using gene sets selected in Step 3 and aggregation algorithm used in Step 4. The diagram abstracts from this operation.

Alternatives considered for factors influencing the set-level learning workflow. The number left of each factor refers to the workflow step (Fig. 2) in which it acts.

*Identified by rank, *n *corresponds to the lowest ranking set, *i*:*j *denotes that all of gene sets ranking *i *to *j *are used to form features.

Given the above, there is no apparent reason why low-ranking gene sets should be used in practical experiments. Therefore, to maintain relevance of the subsequent conclusions, we conducted further analyses on the set-level experimental sample only with measurements where Factor 3 (gene set rank) is either 1 or 1:10.

We next addressed the hypothesis that genuine gene sets constitute better features than random gene sets, i.e. we investigated the influence of Factor 1 in Table 1. Classifiers learned with genuine gene sets exhibited significantly higher predictive accuracies (p = 1.4 × 10^−4^, one-sided test) than those based on random gene sets.

Given this result, there is a clear preference to use genuine gene sets over random gene sets in practical applications. Once again, to maintain relevance of our subsequent conclusions, we constrained further analyses of the set-level sample to measurements conducted with genuine gene sets.

Working now with classifiers learned with high-ranking genuine gene sets, we revisited Factor 3 to assess the difference between the remaining alternatives 1 and 1:10 corresponding respectively to more and less compression of training data. The 1:10 variant where sample features capture information from the ten best gene sets exhibits significantly (p = 3.5 × 10^−5^) higher accuracy than the 1 variant using only the single best gene set to constitute features (that is, a single feature if aggregation is employed).

We further compared the three dedicated gene-set ranking methods, i.e. evaluated the effect of Factor 2 in Table 1. Since three comparisons are conducted in this case (one per pair), we used the Bonferroni-Dunn adjustment on the Wilcoxon test result. The Global test turned out to exhibit significantly higher accuracy than either SAM-GS (p = 0.0051) or GSEA (p = 0.0039). The difference between the latter two methods was not significant.

Concerning the aggregation method (Factor 4 in Table 1), there are two questions of interest: whether there are significant differences in the performance of the individual aggregation methods (SVD, AVG, SetSig), and whether aggregation in general has a detrimental effect on performance. As for the first question, both SVD and SetSig proved to outperform AVG (p = 0.011 and p = 0.03, respectively), while the difference between SVD and SetSig is insignificant. The answer to the second question turned out to depend on Factor 3 as follows. In the more compressive (1) alternative, the answer is affirmative in that all the three aggregation methods result in less accurate classifiers than those not involving aggregation (p = 0.0061 for SVD, p = 0.013 for SetSig and p = 1.1 × 10^−4 ^for AVG, all after Bonferroni-Dunn adjustment).

However, the detrimental effect of aggregation tends to vanish in the less compressive (1:10) alternative of Factor 3, where only the AVG alternative in comparison to None yields a significant difference (p = 0.011). Table 2 summarizes the main findings presented above.

**Table 2:** Summary of results

*Factor*	*Alternatives*	
	*Better*	*Worse*
*1. Gene sets*	Genuine	Random
*2. Ranking algo*	Global	SAM-GS, GSEA
*3. Sets forming features*	high ranking, 1:10 (best ten sets)	low ranking, 1 (best set)
4. Aggregation*	**SetSig**, **SVD**	**AVG**

See Section *Main Results *for details on how the conclusions were determined.

*Difference not significant if Factor 3 is 1:10.

The principal trends can also be well observed through the ranked list of methodological combinations by median classification accuracy, again generated from measurements not involving random or low-ranking gene sets. This is shown in Table 3. Position 17 refers to the baseline method where sample features capture expressions of all genes and prior gene set definitions are ignored. In agreement with the statistical conclusions above, the ranked table clearly indicates the superiority of the Global test for gene-set ranking, and of using the 10 best gene sets (i.e., the 1:10 alternative) to establish features rather than relying only on the single best gene set. It is noteworthy that all four combinations involving the Global test and the 1:10 alternative (i.e., ranks 1, 2, 4, 5) outperform the baseline method.

**Table 3:** Ranking of gene set methods

*Rank*	*Methods*			*Accuracy*			
	*Sets*	*Rank. Algo*	*Aggrgt*	*Median*	*Avg*	*σ*	*Iqr*
1	1:10	Global	SVD	89.2	79.5	18.9	33.2
2	1:10	Global	None	88.3	81.0	17.7	31.3
3	1	Global	None	87.8	80.7	17.5	31.0
4	1:10	Global	SetSig	87.4	81.1	16.5	26.1
5	1:10	Global	AVG	85.6	78.7	18.4	32.6
6	1:10	SAM-GS	SetSig	85.4	79.9	17.1	30.2
7	1:10	SAM-GS	None	84.6	80.1	17.3	30.7
8	1	Global	SVD	83.8	77.9	20.1	34.3
9	1:10	GSEA	SetSig	83.4	78.3	16.7	26.3
10	1:10	GSEA	None	82.3	80.0	16.8	30.4
11	1:10	SAM-GS	SVD	79.9	77.1	18.0	32.1
12	1:10	GSEA	SVD	79.2	77.2	17.7	31.7
13	1:10	GSEA	AVG	79.1	76.4	16.9	31.9
14	1	SAM-GS	None	78.3	76.0	15.3	26.3
15	1	Global	SetSig	77.5	75.9	15.1	23.5
16	1	GSEA	None	76.7	75.6	16.3	29.5
17	*baseline (all genes used)*			75.5	76.6	18.4	33.5
18	1	SAM-GS	SetSig	75.0	74.7	14.2	18.9
19	1	Global	AVG	72.7	73.8	17.6	31.1
20	1:10	SAM-GS	AVG	72.5	73.8	15.9	26.0
21	1	GSEA	SetSig	70.2	72.6	17.0	26.8
22	1	GSEA	AVG	69.6	68.1	12.8	22.4
23	1	GSEA	SVD	69.5	71.9	16.3	28.2
24	1	SAM-GS	SVD	69.0	69.5	15.7	21.3
25	1	SAM-GS	AVG	67.3	67.0	11.4	15.5

Ranking of combinations of gene set methods by median predictive accuracy achieved on 30 datasets (Table 8, Section *Expression and gene sets*) with 5 machine learning algorithms (Section *Machine learning*) estimated through 10-fold cross-validation (i.e. 1,500 experiments per row). The columns indicate, respectively, the resulting rank by median accuracy, the gene sets used to form features (1 - the top ranking set, 1:10 - the top ten ranking sets), the gene set selection method, the expression aggregation method (see Section *Methods and data *for details on the latter 3 factors), and the median, average, standard deviation and interquartile range of the accuracy.

While intuitive, rankings based on median accuracy over multiple datasets may, according to [25], be problematic as to their statistical reliability. Therefore, we offer in Table 4 an alternative ranking of the 19 methods that avoids mixtures of predictive accuracies from different datasets. Here, the methods were sub-ranked on each of the 150 combinations of 30 datasets and 5 learning algorithms by cross-validated predictive accuracy achieved on that combination. The 150 sub-ranks were then averaged for each method, and this average dictates the ranking shown in the table. In this ranking, the baseline strategy improves its rank to Position 5. The superiority of classifiers learned from 10 gene sets selected by the Global test, as formerly noted for Table 3, continues to hold in the alternative ranking underlying Table 4.

**Table 4:** Ranking of all combinations of methods

*Rank*	*Methods *			*Avg Subrank*
	*Sets*	*Rank. algo*	*Aggrgt*	
1	1:10	Global	None	15.3
2	1:10	Global	SetSig	15.7
3	1	Global	None	16.3
4	1:10	GSEA	None	16.7
5	*baseline (all genes used)*			16.8
6	1:10	Global	SVD	17.0
7	1:10	SAM-GS	None	17.2
8	1:10	SAM-GS	SetSig	17.6
9	1:10	Global	AVG	18.6
10	1	Global	SVD	19.4
11	1:10	GSEA	SetSig	19.9
12	1:10	GSEA	SVD	20.1
13	1:10	SAM-GS	SVD	20.8
14	1:10	GSEA	AVG	22.1
15	1	Global	SetSig	22.2
16	1	SAM-GS	None	23.0
17	1	SAM-GS	SetSig	23.8
18	1	GSEA	None	23.9
19	1	Global	AVG	24.6
20	1:10	SAM-GS	AVG	25.5
21	1	GSEA	SVD	26.7
22	1	GSEA	SetSig	26.8
23	1	SAM-GS	SVD	28.3
24	1	SAM-GS	AVG	30.3
25	1	GSEA	AVG	30.9

Ranking of all combinations of methods in terms of average subrank. Subranking is done on each of the 150 combinations of 30 datasets and 5 learning algorithms by cross-validated predictive accuracy. Column descriptions are as in Table 3.

## Additional analyses

### Generic feature selection

In the set-level classification framework, gene sets play the role of sample features. Therefore the three gene-set ranking methods (GSEA, SAM-GS, Global) are employed for feature selection conducted in the learning workflow. While the latter three methods originate from research on gene expression analysis, generic feature selection methods have also been proposed in machine learning research [23]. It is interesting to compare the latter to the gene-expression-specific methods. To this end, we consider two approaches. *Information Gain *(IG) [10] is a feature-selection heuristic popular in machine learning. In brief, IG measures the expected reduction in class-entropy caused by partitioning the given sample set by the values of the assessed feature. One of the main disadvantages of IG is that it disregards potential feature interactions. *Support Vector Machine with Recursive Feature Extraction *(SVM-RFE) [26] is a method that ranks features by repetitive training of a SVM classifier with a linear kernel while gradually removing the feature with the smallest input classifier weight. This approach does not assume that features are mutually independent. On the other hand, it naturally tends to select a feature set that maximizes the accuracy of the specific kind of classifier (SVM). For computational reasons (large number of runs and genes), we removed several features at a time (*F *× 2^−*i *^features in the *i*-th iteration, where *F *is the original number of features). [26] mentions such a modification with the caveat that it may be at the expense of possible classification performance degradation.

In the present context, generic feature selection can be applied either on the gene level or on the set level. We explored both scenarios.

The gene-level application produces a variant of the baseline classifier (position 17 in Table 3, position 5 in Table 4) where, however, the learning algorithm only receives features corresponding to genes top-ranked by the feature selection heuristic, rather than all measured genes. The selection is thus based only on the predictive power of the individual genes and ignores any prior definitions of gene sets. The question of how many top-ranking genes should be used for learning is addressed as follows. We want to make the resulting predictive accuracy comparable to that obtained in the main (set-level) experimental protocol, in particular to the 1 and 1:10 alternatives of Factor 3. The median of the number of unique genes present in the selected gene sets in the 1 (1:10, respectively) alternative is 22 (228). Therefore we experiment respectively with 22 and 228 genes top-ranked by generic feature selection. The results are shown in Table 5. Comparing the latter to Tables 3 and 4, we observe that both variants improve the baseline and in fact produce the most accurate classifiers (IG outperforms the set-level approaches, SVM-RFE is comparable with the Global test). SVM-RFE does not outperform IG in general, but it does so in the special case when SVM is used as the learning algorithm.

**Table 5:** Generic feature selection (gene-level)

*# Method*	*# Selected Genes*	*Accuracy*				*Avg Subrank*
		*Median*	*Avg*	*σ*	*Iqr*	
IG	22	90.2	81.5	18.1	30.7	15.0
IG	228	89.8	82.0	17.9	30.3	14.5
SVM-RFE	228	88.3	82.3	16.7	28.5	16.4
SVM-RFE	22	88.0	82.1	17.2	30.4	16.2

Performance of the baseline classification method equipped with a feature-selection step prior to learning. Features (genes) are ranked by the information gain and SVM-RFE heuristics. The number of selected top-ranking genes (22 and 228, respectively) corresponds to the mean number of unique genes acting in gene sets selected in the 1 and 1:10 (respectively) alternatives of the set-level workflow.

While the gene-level application of feature selection results in accurate classifiers, the obvious drawback of this approach is that the genes referred in such produced classifiers cannot be jointly characterized by a biological concept. This deficiency is removed if feature selection is instead applied on the set level, i.e. to rank apriori-defined gene sets. This way, the selection methods essentially become the fourth and fifth alternative of Factor 2 (see Table 1) up to the following nuance. While the dedicated gene-set methods (GSEA, SAM-GS, Global) score a feature (gene set) by the expressions of its multiple member genes, IG and SVM-RFE score a feature by the single real value assigned to it, i.e., by the aggregated expressions of the member genes. Therefore, when using the generic feature selection, the aggregation step in the experimental workflow (Figure 2) must precede the ranking step. The results of applying IG and SVM-RFE on the set level are shown in Table 6. Comparing again to Tables 3 and 4, both IG and SVM-RFE are outperformed by the Global test (Wilcoxon test, p = 0.017).

**Table 6:** Generic feature selection (set-level)

*Sets*	*Methods*		*Accuracy*				*Avg Subrank*
	*Selection*	*Aggrgt*	*Median*	*Avg*	*σ*	*Iqr*	
1:10	SVM-RFE	SVD	88.3	80.6	17.3	33.0	17.6
1:10	IG	SVD	87.0	79.0	18.7	31.6	17.4
1:10	IG	AVG	84.6	78.2	18.6	33.4	18.7
1:10	SVM-RFE	AVG	84.4	79.2	17.1	31.2	19.2
1:10	SVM-RFE	SetSig	82.5	78.7	17.0	31.2	19.4
1	IG	SVD	80.8	76.3	17.7	33.1	22.5
1:10	IG	SetSig	80.0	77.1	17.4	33.2	20.8
1	SVM-RFE	SetSig	71.8	73.7	15.8	26.4	23.3
1	SVM-RFE	SVD	71.5	74.4	17.4	30.3	23.0
1	IG	AVG	70.9	74.0	18.6	33.1	24.1
1	SVM-RFE	AVG	70.8	72.5	15.4	26.6	24.4
1	IG	SetSig	66.2	68.8	16.2	25.0	28.9

Performance of the set level classification strategy using the information gain and SVM-RFE heuristics for ranking gene sets. Column descriptions are as in Table 3.

### Successful gene sets

We also explored patterns distinguishing gene sets particularly useful for classification from other employed gene sets sourced from the Molecular Signatures Database. To this end, we defined three groups of gene sets. The first group referred to as *full *comprises the entire collection of 3028 gene sets obtained from the database (gene sets containing fewer than 5 or more than 200 genes were discarded). The second group referred to as *selected *consists of the 900 gene sets ranked high (1^st ^to 10^th^) by any of the three selection methods for any of the dataset. The third group referred to as *successful *is a subset of the *selected *group and contains the 210 gene sets acting in classifiers that outperformed the baseline.

We investigated two kinds of properties of the gene sets contained in the three respective groups. First, we considered the gene set type as defined in the Molecular Signatures Database. The gene sets belonging to the category of chemical and genetic perturbations (CGP) were more frequently *selected *and also more frequently appeared in the *successful *group than the gene sets representing canonical pathways (CP) (full: CGPs 73%, CPs 27%, selected: CGPs 88%, CPs 12%, successful: CGPs 88%, CPs 12%). Second, we considered four possible notions of gene set *size*: i) nominal size (the gene set cardinality), ii) effective size (number of genes from the gene set measured in the dataset), iii) number of PCA coefficients capturing 50% of expression variance in the gene set, iv) as in iii) but with 90% variance. As follows from Table 7, the *successful *group contains smaller gene sets than the other two groups, and this trend is most pronounced for the Global test ranking method (Mann-Whitney U test, the *successful *group versus the *full *group, Bonferroni adjustment: Effective size p = 0.084, PCA 90% p = 0.0039).

**Table 7:** Comparison of the full, selected and successful group of gene sets

*Group*	*Selection*	*Statistic*	*Nominal size*	*Effective size*	*PCA 50% var*	*PCA 90% var*
*Full*	None	mean	71.7±1.7	40.9±0.7	4.4±0.03	16.7±0.14

		median	37.0	28.1	4.1	15.3
*Selected*	all	mean	62.5±2.7	47.8±1.9	3.8±0.08	15.1±0.35
		median	33.5	27.0	3.4	13.4
	Global	median	32.0	25.5	3.3	12.8
	GSEA	median	34.0	27.0	3.4	13.7
	SAM-GS	median	40.5	28.0	3.7	14.3

*Successful*	all	mean	56.9±4.4	39.2±2.9	4.3±0.14	14.7±0.56
		median	31.0	21.0	3.9	12.6
	Global	median	22.0	18.5	3.8	11.7
	GSEA	median	37.0	27.5	4.3	14.2
	SAM-GS	median	30.5	22.5	4.0	12.7

Mean and median sizes of gene sets partitioned into three groups (see Section *Successful gene sets *for details.)

## Conclusions and discussion

Set-level approaches to gene expression data analysis have proliferated in the last years, evidence of which are both theoretical studies [1,2] and software tools with set-level functionalities [27] such as enrichment analysis. The added insight and augmented interpretability of analysis results are the main reasons for the popularity of the set-level framework. For the same reasons, the framework has recently been also explored in the context of predictive classification of gene expression data through machine learning [4,17-22]. Conclusions of such studies have however been rather limited as to the range of classification problems considered and techniques used in the set-level machine learning workflow, and inconclusive as to the statistical performance of set-level classifiers. To this end, we have presented a large experimental study, in which we formalized the mentioned set-level workflow, identified various independently published techniques relevant to its individual steps, and reformulated them into a unified framework. By executing various instantiations of the workflow on 30 gene expression classification problems, we have established the following main conclusions.

1. State-of-the-art gene set ranking methods (GSEA, SAM-GS, Global test) perform sanely as feature selectors in the machine learning context in that high ranking gene sets outperform (i.e., constitute better features for classification than) those low ranking.

2. Genuine curated gene sets from the Molecular Signature Database outperform randomized gene sets. Smaller gene sets and sets pertaining to chemical and genetic perturbations were particularly successful.

3. For gene set selection, the Global test [2] outperforms each of SAM-GS [3], GSEA [1] as well as the generic information gain heuristic [10] and the SVM-based recursive feature elimination approach [26].

4. For aggregating expressions of set member genes into a unique feature value, both SVD [7] and SetSig [22] outperform arithmetic averaging [4].

5. Using top ten gene sets to construct features results in better classifiers than using only the single best gene set.

6. The set-level approach using top ten genuine gene sets as ranked by the Global test outperforms the baseline gene-level method in which the learning algorithm is given access to expressions of all measured genes. However, it is outperformed by the baseline approach if the latter is equipped with a prior feature selection step.

Conclusion 1 is rather obvious and was essentially meant as a prior sanity check.

The first statement of Conclusion 2 is not obvious, since constructing randomized gene sets in fact corresponds to the machine learning technique of stochastic feature extraction [28] and as such may itself contribute to learning good classifiers. Nevertheless, relevant background knowledge resting in the prior definition of biologically plausible gene sets contributes further to increasing the predictive accuracy. Conclusions 3 and 4 are probably the most significant for practitioners in set-level predictive modeling of gene expression as so far there has been no clear guidance to choose from the two triples of methods.

Concerning Conclusion 3, the advantages of the Global test were argued in [2] but not supported in terms of the predictive power of the selected gene sets. As for conclusion 4, the SetSig technique was introduced and tested in [22], appearing superior to both averaging and a PCA-based method which is conceptually similar to the SVD method [7]. However, owing to the limited experimental material in [22], the ranking was not confirmed by a statistical test. Here we confirmed the superiority of SetSig with respect to averaging, however, the difference of in the performance of SetSig and SVD was not significant.

A further remark concerns the mentioned aggregation methods. All three of them are applicable to any kind of gene sets, whether these are derived from pathways, gene ontology or other sources of background knowledge. The downside of this generality is that substantial information available for specific kinds of gene sets is ignored. Of relevance to pathway-based gene sets, the recent study [29] convincingly argues that the perturbation of a pathway depends on the expressions of its member genes in a non-uniform manner. It also proposes how to quantify the impact of each member gene on the perturbation, given the graphical structure of the pathway. It seems reasonable that a pathway-specific aggregation method should also weigh member genes by their estimated impact on the pathway. Such a method would likely result in more informative pathway-level features and could outperform the three aggregation methods we have considered.

**Figure 3:**
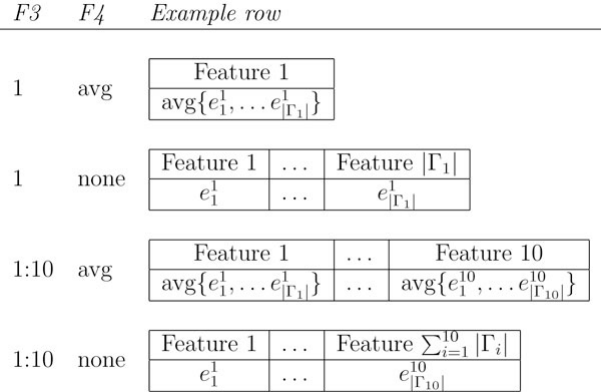
**Examples of sample representation**. Examples of sample representation generated with four combinations of alternatives of factors 3 and 4 from Table 1. Shown for one sample (i.e. header + one row) with $e_{i}^{j}$ denoting the expression of the *i*-th member of the *j*-ranked gene set Γ*_j_*. Non-exemplified combinations of the two factors are analogical to the cases shown. The remaining considered factors do not influence the structure of sample representation.

Conclusion 5 is not entirely surprising. Relying only on a single gene set entails too large an information loss and results in classifiers less accurate than those using ten best gene sets. Note that in the single gene set case, when aggregation is applied (i.e., Factor 4 in Table 1 is other than None, see the first example in Figure 3), the sample becomes represented by only a single real-valued feature and learning essentially reduces to finding a threshold value for it. To verify that more than one gene set should be taken into account, we tested the 10-best-sets option and indeed it performed better. Obviously, the optimal number of sets to be considered depends on the particular classification problem and data, and in practice it can be estimated empirically, e.g. through internal cross-validation. Here, training data *T *would be randomly split into a validation set *V *and the remainder *T*' = *T *\ *V *, e.g. with the 20%-80% proportion. Classifiers would first be learned with *T*', each with a different value for the number of gene sets forming features; this number could range e.g. as *f *∈ {2, 4, 8,..., 128}. The number f* yielding the classifier most accurate on the validation set *V *is then an estimate of the optimal number of features. The final classifier would then be learned on the entire training set *T*, using f* features. While we could not follow this procedure due to computational considerations (the already high number of learning sessions would have grown excessively), it is a reasonable instrument in less extensive experiments such as in single-domain classification.

A straightforward interpretation of Conclusion 6 is that the set-level framework is not an instrument for boosting predictive accuracy. However, set-level classifiers have a value per se, just as set-level units are useful in standard differential analysis of gene expression data. In this light, it is important that with a suitable choice of techniques, set-level classifiers do achieve accuracy competitive with conventional gene-level classifiers.

## Methods and data

Here we first describe the methods adopted for gene set ranking, gene expression aggregation, and for classifier learning. Next we present the datasets used as benchmarks in the comparative experiments. Lastly, we describe the protocol followed by our experiments.

### Gene set ranking

Three methods are considered for ranking gene sets. As inputs, all of the methods take a set *G *= {*g*_1_, *g*_2_,...*g_p_*} of interrogated genes, and a set *S *of *N *expression samples where for each *s_i _*∈ S, *s_i _*= (*e*_1,*i*_, *e*_2,*i*_,...*e_p,i_*) ∈ R*^p ^*where *e_j,i _*denotes the (normalized) expression of gene *g_j _*in sample *s_i_*. The sample set *S *is partitioned into phenotype classes *S *= *C*_1_∪*C*_2_∪...∪*C_o _*so that *C_i_*∩*C_j_*={} for *i *≠ *j*. To simplify this paper, we assume binary classification, i.e. *o *= 2. A further input is a collection of gene sets  such that for each Γ∈G it holds Γ ⊆ *G*. In the output, each of the methods ranks all gene sets in  by their estimated power to discriminate samples into the predefined classes.

Next we give a brief account of the three methods and refer to the original sources for a more detailed description. In experiments, we used the original implementations of the procedures as provided or published by the respective authors.

**Gene Set Enrichment Analysis (GSEA) **[1] tests a null hypothesis that gene rankings in a gene set Γ, according to an association measure with the phenotype, are randomly distributed over the rankings of all genes. It first sorts *G *by correlation with binary phenotype. Then it calculates an enrichment score (ES) for each Γ∈G by walking down the sorted gene list, increasing a running-sum statistic when encountering a gene *g_i _*∈ Γ and decreasing it otherwise. The magnitude of the change depends on the correlation of *g_i _*with the phenotype. The enrichment score is the maximum deviation from zero encountered in the random walk. It corresponds to a weighted Kolmogorov-Smirnov-like statistic. The statistical significance of the ES is estimated by an empirical phenotype-based permutation test procedure that preserves the correlation structure of the gene expression data. GSEA was one of the first specialized gene-set analysis techniques. It has been reported to attribute statistical significance to gene sets that have no gene associated with the phenotype, and to have less power than other recent test statistics [2,3].

#### SAM-GS [3]

This method tests a null hypothesis that the mean vectors of the expressions of genes in a gene set do not differ by phenotype. Each sample *s_i _*is viewed as a point in an *N *-dimensional Euclidean space. Each gene set Γ∈G defines its |Γ|-dimensional subspace in which projections $s_{i}^{\Gamma }$ of samples *s_i _*are given by coordinates corresponding to genes in Γ. The method judges a given by how distinctly the clusters of points $\left\{s_{i}^{\Gamma }|s_{i}\in C_{1}\right\}$ and $\left\{s_{j}^{\Gamma }|s_{j}\in C_{2}\right\}$ are separated from each other in the subspace induced by Γ. SAM-GS measures the Euclidean distance between the centroids of the respective clusters and applies a permutation test to determine whether, and how significantly, this distance is larger than that obtained if samples were assigned to classes randomly.

#### The Global Test [2]

The global test, analogically to SAM-GS, projects the expression samples into subspaces defined by gene sets Γ∈G. In contrast to the Euclidean distance applied in **SAM-GS**, it proceeds instead by fitting a regression function in the subspace, such that the function value acts as the class indicator. The degree to which the two clusters are separated then corresponds to the magnitude of the coefficients of the regression function.

### Expression aggregation

Three methods are considered for assigning a value to a given gene set Γ for a given sample *s_i _*by aggregation of expressions of genes in Γ.

#### Averaging (AVG)

The first method simply produces the arithmetic average of the expressions *e_j,i _*of all Γ genes 1 ≤ *j *≤ *p *in sample *s_i_*. The value assigned to the pair (*s_i_*, Γ) is thus independent of samples *s_j_*, *i *≠ *j*.

#### Singular Value Decomposition (SVD)

A more sophisticated approach was employed by [7]. Here, the value assigned to (*s_i_*, Γ) depends on expressions *e_j,i _*measured in sample *s_i _*but, unlike in the averaging case, also on expressions *e_j,k _*measured in samples *s_k_*, *k *≠ *i*. In particular, all samples in the sample set *S *are viewed as points in the |Γ|-dimensional Euclidean space induced by Γ the same way as explained in Section *Gene set ranking*. Subsequently, the specific vector in the space is identified, along which the sample points exhibit maximum variance. Each point *s_k _*∈ *S *is then projected onto this vector. Finally, the value assigned to (*s_i_*, Γ) is the real-valued position of the projection of *s_i _*on the maximum-variance vector in the space induced by Γ.

#### Gene Set Signatures (SetSig)

Similarly to the SVD method, the SetSig [22] method assigns to (*s_i_*, Γ) a value depending on expressions both in sample *s_i _*as well as in other samples *s_k_*, *k *≠ *i*. However, unlike in the previous two aggregation methods, here the value also depends on the class memberships of these samples. In particular, SetSig confines to two-class problems and the value ('signature') assigned to (*s_i_*, Γ) can be viewed as the Student's unpaired t-statistic for the means of two populations of the Pearson correlation coefficients. The first (second) population studies correlation of *s_i _*with the samples from the first (second) class in the space induced by Γ. Intuitively, the signature is positive (negative) if the sample correlates rather with the samples belonging to the first (second) class.

### Machine learning

We experimented with five diverse machine learning algorithms to avoid dependence of experimental results on a specific choice of a learning method. These algorithms are explained in depth for example by [8]. In experiments, we used the implementations available in the WEKA software due to [30], using the default settings. None of the methods below is in principle superior to the others, although the first one prevails in predictive modeling of gene expression data and is usually associated with high resistance to noise in data.

#### Support Vector Machine

Samples are viewed as points in a vector space with coordinates given by the values of its features. A classifier is sought in the form of a hyperplane that separates training samples of distinct classes and maximizes the distance to the points nearest to the hyperplane (i.e. maximizing the *margin*) in that space or in a space of extended dimension into which the original vector space is non-linearly projected.

#### 1-Nearest Neighbor

This algorithm is a simple form of classification proceeding without learning a formal data model. A new sample is always predicted to have the same class as the most similar sample (i.e. the nearest neighbor) available in training data. We use the Euclidean metric to measure the similarity of two samples.

#### 3-Nearest Neighbors

This method is similar to 1-Nearest Neighbor, except that class is determined as one prevailing among the three, rather than one, most similar samples in training data. This method becomes superior to the previous one as noise in data exceeds a certain threshold amount. The threshold value (and thus the optimal number of considered neighbors) is in general not known.

#### Naive Bayes

A sample is classified into the class that is most probable given the sample's feature values, according to a conditional probability distribution learned from training data on the simplifying assumption that, within each class, all features are mutually independent random variables. Gene expression data usually deviate from this assumption and consequently the method becomes suboptimal.

#### Decision Tree

A tree-graph model enables to derive a class prediction for a sample by following a path from the root to a leaf of the tree, where the path is determined by outcomes of tests on the values of features specified in the internal nodes of the tree. The tree model is learned from training data and can also be represented as a set of decision rules.

### Expression and gene sets

**Figure 4:**
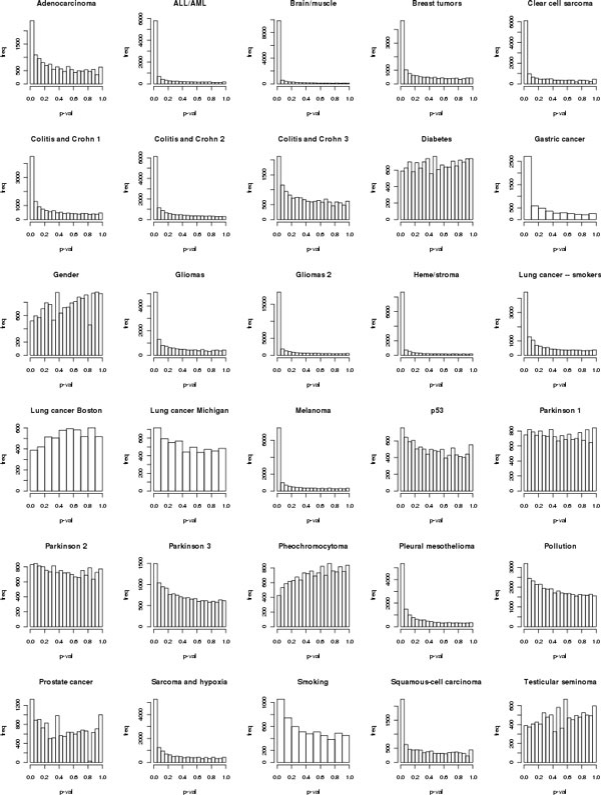
**Histograms of differential gene expression**. Histograms of differential gene expression suggest the difficulty of the individual domains. An easy domain is supposed to have a strongly left-skewed histogram, while the difficult domains rather show a flat histogram. There is one plot for each of 30 domains, *x *axis shows the p-value of differential expression, the *y *axis gene frequency.

We conducted our experiments using 30 public gene expression datasets, each containing samples categorized into two classes. This collection contains both hard and easy classification problems (see Figure 4). The individual datasets are listed in Table 8 and annotated in more detail in the supplemental material at http://ida.felk.cvut.cz/CESLT.

**Table 8:** Datasets

*Dataset*	*Genes*	*Class 1*	*Class 2*	*Source*	*Reference*
Adenocarcinoma	14023	8	29	GDS2201	[31]
ALL/AML	10056	24	24	Broad institute	[32]
Brain/muscle	13380	41	20	-	[4]
Breast tumors	14023	16	27	GDS1329	[33]
Clear cell sarcoma	14023	18	14	GDS1282	[34]
Colitis and Crohn 1	14902	42	26	GDS1615	[35]
Colitis and Crohn 2	14902	42	59	GDS1615	[35]
Colitis and Crohn 3	14902	26	59	GDS1615	[35]
Diabetes	13380	17	17	Broad institute	[5]
Heme/stroma	13380	18	33	-	[4]
Gastric cancer	5664	8	22	GDS1210	[36]
Gender	15056	15	17	Broad institute	[1]
Gliomas	14902	26	59	GDS1975	[37]
Gliomas 2	31835	23	81	GDS1962	[38]
Lung cancer Boston	5217	31	31	Broad institute	[39]
Lung cancer Michigan	5217	24	62	Broad institute	[40]
Lung cancer - smokers	14023	90	97	GDS2771	[41]
Melanoma	14902	18	45	GDS1375	[42]
p53	10101	33	17	Broad institute	[1]
Parkinson 1	14902	22	33	GDS2519	[43]
Parkinson 2	14902	22	50	GDS2519	[43]
Parkinson 3	14902	33	50	GDS2519	[43]
Pheochromocytoma	14023	38	37	GDS2113	[44]
Pleural mesothelioma	14902	10	44	GDS1220	[45]
Pollution	37804	88	41	-	[46]
Prostate cancer	14023	18	45	GDS1390	[47]
Sarcoma and hypoxia	14902	15	39	GDS1209	[48]
Smoking	5664	18	26	GDS2489	[49]
Squamous-cell carcinoma	9460	22	22	GDS2520	[50]
Testicular seminoma	9460	22	14	GDS2842	[51]

Number of genes interrogated and number of samples in each of the two classes of each dataset.

Besides expression datasets, we utilized a gene set database consisting of 3272 manually curated sets of genes obtained from the Molecular Signatures Database (MSigDB v3.0) [1]. These gene sets have been compiled from various online databases (e.g. KEGG, GenMAPP, BioCarta).

For control experiments, we also prepared another collection of gene sets that is identical to the latter in the number of contained sets and the distribution of their cardinalities. However, the contained sets are assembled from random genes and have no biological significance. The particular method used to obtain the randomized gene sets is as follows. For sampling, we consider the set Σ of all genes occurring in some of the genuine gene sets, formally Σ={g|g∈Γ,Γ∈G}. Then, for each genuine gene set Γ, we sample |Γ| genes without replacement uniformly from Σ to constitute the counterpart random gene set Γ'.

### Experimental protocol

Classifier learning in the set-level framework follows a simple workflow. Its performance is influenced by several factors, each corresponding to a particular choice from a class of techniques (such as for gene set ranking). We evaluate the contribution that these factors make to the predictive accuracy of the resulting classifiers by repeated executions of the learning workflow with varying the factors.

The learning workflow is shown in Figure 2. Given a set of binary-labeled training samples from an expression dataset, the workflow starts by ranking the provided collection of a priori-defined gene sets according to their power to discriminate sample classes. The resulting ranked list is subsequently used to select the gene sets which form set-level sample features. Each such feature is then assigned a value for each training sample by aggregating the expressions in the gene set corresponding to the feature. An exception to this pattern is the *None *alternative of the aggregation factor, where expressions are not aggregated, and features correspond to genes instead of gene sets. This alternative is considered for comparative purposes. Figure 3 illustrates the resulting sample representation for four combinations of the selection and aggregation alternatives. Next, a machine learning algorithm produces a classifier from the reformulated training samples. Finally, the classifier's predictive accuracy is calculated as the proportion of samples correctly classified on an independent testing sample fold. For compatibility with the learned classifier, the testing samples are also reformulated to the set level prior to testing, using the same selected gene sets and aggregation mechanism as in the training phase.

Seven factors along the workflow influence its result. The alternatives considered for each of them are summarized in Table 1. We want to assess the contributions of the first four factors (top in table). The remaining three auxiliary factors (bottom in table) are employed to diversify the experimental material and thus increase the robustness of the findings. Factor 7 (testing fold) is involved automatically through the adoption of the 10-fold cross-validation procedure (see e.g. chap. 7 in [8]). We execute the workflow for each possible combination of factor alternatives, obtaining a factored sample of 792,000 predictive accuracy values.

While the measurements provided by the above protocol allow us to compare multiple variants of the set-level framework for predictive classification, we also want to compare these to the baseline gene-level alternative usually adopted in predictive classification of gene expression data. Here, each gene interrogated by a microarray represents a feature. This sample representation is passed directly to the learning algorithm without involving any of the pre-processing factors (1-4 in Table 1). The baseline results are also collected using the 5 different learning algorithms, the 30 benchmark datasets and the 10-fold cross-validation procedure (i.e. Factors 5-7 in Table 1 are employed). As a result, an additional sample of 1,500 predictive accuracy values is collected for the baseline variant.

Finally, to comply with the standard application of the cross-validation procedure, we averaged the accuracy values corresponding to the 10 cross-validation folds for each combination of the remaining factors. The subsequent statistical analysis thus deals with a sample of 79,200 and 150 measurements for the set-level and baseline experiments, respectively, described by the predictive accuracy value and the values of the relevant factors.

All statistical tests conducted were based on the paired Wilcoxon test (two-sided unless stated otherwise). For pairing, we always related two measurements equal in terms of all factors except for the one investigated. The stronger t-test is more usual in analysis of predictive accuracy samples in literature but our preliminary normality tests did not justify its application. Given the extent of the collected samples, the Wilcoxon test was sufficient to support the conclusions reported. Besides, the Wilcoxon test is argued [25] to be statistically safer than the t-test for comparing classification algorithms over multiple data sets.

## Competing interests

The authors declare that they have no competing interests.

## Authors' contributions

MH collected the experimental data, implemented the experimental framework and accomplished the experiments. JK carried out the statistical evaluation of the study and partly wrote the manuscript. JK and FZ co-designed the experimental framework. FZ supervised all steps of the work and conceived the paper. JT motivated the initial phases of the study and revised the manuscript. All the authors read and approved the manuscript.
